# Anesthetic-sensitive ion channel modulation is associated with a molar water solubility cut-off

**DOI:** 10.1186/s40360-018-0244-z

**Published:** 2018-09-14

**Authors:** Robert J. Brosnan, Trung L. Pham

**Affiliations:** Department of Surgical and Radiological Sciences, School of Veterinary Medicine, University of California, One Shields Avenue, Davis, CA 95616 USA

**Keywords:** Anesthesia, Mechanism, Ion channel, Electrophysiology, Aliphatic

## Abstract

**Background:**

NMDA receptor modulation by hydrocarbons is associated with a molar water solubility cut-off. Low-affinity phenolic modulation of GABA_A_ receptors is also associated with a cut-off, but at much lower molar solubility values. We hypothesized that other anesthetic-sensitive ion channels exhibit distinct cut-off effects associated with hydrocarbon molar water solubility, and that cut-off values are comparatively similar between related receptors than phylogenetically distant ones.

**Methods:**

Glycine or GABA_A_ receptors or TREK-1, TRESK, Na_v_1.2, or Na_v_1.4 channels were expressed separately in frog oocytes. Two electrode voltage clamp techniques were used to study current responses in the presence and absence of hydrocarbon series from eight functional groups with progressively increasing size at saturated aqueous concentrations. Null response (cut-off) was defined by current measurements that were statistically indistinguishable between baseline and hydrocarbon exposure.

**Results:**

Ion channels exhibited cut-off effects associated with hydrocarbon molar water solubility in the following order of decreasing solubility: Na_v_1.2 ≈ Na_v_1.4 ≳ TRESK ≈ TREK-1 > GABA_A_ >> glycine. Previously measured solubility cut-off values for NMDA receptors were intermediate between those for Na_v_1.4 and TRESK.

**Conclusions:**

Water solubility cut-off responses were present for all anesthetic-sensitive ion channels; distinct cut-off effects may exist for all cell surface receptors that are sensitive to volatile anesthetics. Suggested is the presence of amphipathic receptor sites normally occupied by water molecules that have dissociation constants inversely related to the cut-off solubility value. Poorly soluble hydrocarbons unable to reach concentrations sufficient to out-compete water for binding site access fail to modulate the receptor.

## Background

Protein-ligand interactions are commonly described using a “lock-and-key” model in which the protein and ligand have to fit together to have a chemical effect [1]. Although inhaled anesthetics are presumed to exert their effects through ion channels and cell surface receptor proteins, the diverse nature of both anesthetic ligands and the proteins they modulate challenge assumptions of a conserved complementary structure [2, 3]. Current and historical inhaled anesthetics include single atoms, triatomic molecules, various alkanes (with and without halogens), various ethers (with and without halogens), and various alkenes (with and without halogens); experimental inhaled anesthetics demonstrate even greater structural variety. Hence, these drugs act as structurally diverse keys that each can open the same lock. Moreover, a single inhaled anesthetic can allosterically modulate function of a large number of structurally diverse and phylogenetically unrelated ion channels—including many different ligand-gated ion channels, voltage-gated ion channels, and leak channels—and cell surface receptors—including many different channel-linked receptors, enzyme-linked receptors, and G protein-coupled receptors. Hence, these agents also act like a key that can open many different locks.

Inhaled anesthetics typically have high median effective concentrations consistent with low-affinity target interactions [4], and these agents can bind multiple amphipathic sites on a protein of which at least some may be associated with water molecules [5]. Anesthetic modulation of protein function firstly depends upon the presence of an agent in sufficient concentration to desolvate, displace weakly-bound water molecules within an allosteric binding site, and only then bind to the allosteric site. We postulate that drugs with insufficient water solubility to competitively displace water from this amphipathic allosteric site would be unable to bind and modulate the protein—a cut-off effect—even at a saturated aqueous phase drug concentration. These low-affinity amphipathic drug-receptor interactions could be associated with different hydrocarbon solubility cut-off values for different proteins since the water dissociation constants within critical allosteric sites could also be different for different proteins. In support, N-methyl-d-aspartate (NMDA) receptor whole-cell currents are unchanged by saturated concentrations of organic compounds once the hydrocarbon ω-end or ring size is increased such that the predicted molar water solubility is less than approximately 1.1 mM; yet these same hydrocarbons still are able to allosterically modulate γ-aminobutyric acid receptor type A (GABA_A_) currents [6]. However, GABA_A_ receptors may have their own hydrocarbon cut-off, as GABA_A_ receptor whole-cell currents are unchanged by a series of substituted phenol and benzene rings once the predicted molar water solubility of these compounds is less than 0.1 mM [7].

We hypothesize that cut-off effects associated with molar water solubility is a generalizable feature of inhaled anesthetic-sensitive ion channels and receptors. As a test, we evaluated responses of anesthetic-sensitive channels and receptors to eight different functional groups of organic compounds, differing only by carbon additions to the ω-end of a chain or to a ring, so that we could study compounds of different molecular volumes and carbon atoms but similar molar water solubility values (and vice versa) and therefore distinguish between effects caused by drug size versus solubility. Although inhaled anesthetics bind many different proteins, we studied six channels and receptors reported to contribute to immobilizing effects in vivo: voltage-gated sodium channels type II and type IV (Na_v_1.2 and Na_v_1.4), [8] TWIK-related spinal cord channel (TRESK) [9], TWIK-related potassium channel type I (TREK-1) [10], GABA_A_ receptors [11], and glycine receptors [12].

## Methods

### Oocyte collection

Adult female *Xenopus laevis* vivarium-maintained frogs (Xenopus Express, Brooksville, FL) were anesthetized with chilled 0.2% buffered tricaine, surgically ovariectomized, and were either administered morphine analgesia and recovered from anesthesia (first ovariectomy) or euthanized (second ovariectomy) by decapitation and double pithing while anesthetized. The theca externa and mesovarium of the removed ovary were disrupted manually, and oocytes were defolliculated enzymatically by use of 0.2% collagenase. Oocytes were stored in a modified Barth’s electrolyte solution until ready for use. This protocol was approved by the Institutional Animal Care and Use Committee at the University of California, Davis.

### Receptor expression

Each anesthetic-sensitive receptor type was separately expressed in different oocytes by injecting plasmids containing sequences for receptor subunits which were provided as gifts from the laboratory of R. Adron Harris (University of Texas, Austin). All plasmids were sequenced and their identity confirmed by comparison to National Center for Biotechnology Information databases. Human voltage-gated Type II sodium channels (Na_v_1.2) were expressed by intracytoplasmic injection of 5 ng RNA per oocyte containing the SCN2A gene; human voltage gated Type IV sodium channels (Na_v_1.4) were similarly expressed using RNA encoding the SCN4A gene. The human TRESK channel (K_2P_18.1) was expressed by intracytoplasmic injection of 5 ng RNA per oocyte containing the KCNK18 gene; the human TREK-1 channel (K_2P_2.1), was similarly expressed using RNA encoding the KCNK2 gene. Heterotrimeric GABA_A_ receptors were expressed by a 1 ng oocyte intranuclear co-injection of three different plasmids containing cytomegalovirus promoters and coding DNA for one of thee subunits—human α_1_ (GABRA1), rat β_2_ (GABRB2), or rat γ_2s_ (GABRG2 short)—in a 1:1:10 ratio to ensure incorporation of the γ-subunit; this was confirmed by demonstrating receptor potentiation to 10 μM chlordiazepoxide during co-application with GABA. Glycine receptors were expressed by intracytoplasmic injection of 5 ng RNA per oocyte containing the gene for the human glycine α_1_ subunit (GLRA1). Nuclease-free water-injected oocytes served as negative controls for all experiments.

### Electrophysiology studies

Oocytes were incubated at 18 °C for 1–4 days in fresh and filtered modified Barth’s solution composed of 88 mM NaCl, 1 mM KCl, 2.4 mM NaHCO3, 20 mM HEPES, 0.82 mM MgSO_4_, 0.33 mM Ca(NO_3_)_2_, 0.41 mM CaCl_2_, 5 mM sodium pyruvate, gentamycin, penicillin, streptomycin, and corrected to pH = 7.4. All salts and antibiotics were A.C.S. grade (Fisher Scientific, Pittsburgh, PA). Oocytes were studied in a 250 μL linear-flow chamber perfused with frog Ringer’s (FR) solution composed of 115 mM NaCl, 2.5 mM KCl, 1.8 mM CaCl_2_, and 10 mM HEPES prepared in 18.2 MΩ H2O and filtered and adjusted to pH = 7.4. All solutions were delivered to the perfusion chamber via a syringe pump with gastight glass syringes and foil-wrapped Teflon tubing.at a rate of 1.5 ml/min. Oocytes were impaled by two 3 M KCl-filled 0.2–1 MΩ borosilicate glass electrodes (KG-33, King Precision Glass, Claremont, CA) connected to separate headstages (Axon Instruments HS2A, Molecular Devices, San Jose, CA) through which voltage was measured and current was passed by use of a computer-controlled amplifier (Axon Instruments GeneClamp 500B, Molecular Devices, San Jose, CA) [6].

For oocytes expressing Na_v_ channels, the cell transmembrane potential was clamped at -80 mV with a 200 ms step increase to 0 mV applied approximately every minute. The Na_v_ channel response was calculated as the difference between the current required to maintain the holding potential and the largest negative current deflection produced by the voltage step. For oocytes expressing K_2P_ channels, the transmembrane potential was clamped at −60 mV, and voltage was stepped to + 60 mV each minute. The K_2P_ channel response was calculated as the difference between holding potential current and voltage step plateau current. For both channel types, baseline responses were measured during oocyte perfusion with FR for a minimum of three times to demonstrate response variability <10%. The perfusate was then switched to the hydrocarbon test drug dissolved at a saturated aqueous phase concentration in FR for 2 min after which the current response to the step voltage was measured. The hydrocarbon drug was then washed out for 5–10 min, and the current response was again measured to demonstrate that it was within 10% of the initial baseline response.

For two-electrode voltage clamp studies in oocytes expressing GABA_A_ receptors or glycine receptors, the transmembrane potential was clamped at −80 mV while being perfused with FR. Approximately every 5 min, the perfusate was switched for 30 s to FR + EC_10_ agonist—20 μM 4-aminobutanoic acid for GABA_A_ receptors or 40 μM aminoethanoic acid for glycine receptors—and then the perfusate was switched back to FR for agonist washout. The current response for each ligand-gated ion channel was calculated as the difference between the peak current measured in the presence of agonist and the whole-cell current measured immediately prior to agonist exposure. Baseline responses were measured in triplicate to confirm <10% variability of current responses in the absence of drug exposure. Next, the perfusate was switched to the hydrocarbon test drug dissolved at a saturated aqueous phase concentration for 2 min followed by a 30s exposure the same concentration of hydrocarbon dissolved in FR + agonist. The hydrocarbon drug was then washed out, and oocytes were exposed to FR + agonist 5 and 10 min later to verify return of the current response to within 10% of pre-drug baseline levels.

### Drug solution preparation

Using predicted density values published in SciFinder (Chemical Abstracts Service, American Chemical Society, Columbus OH), drug solutions were prepared by anaerobic addition of a hydrocarbon volume to gastight glass syringes containing either FR or FR + agonist to create a concentration equal to its calculated molar water solubility (Table 1). After vigorous agitation, an immiscible hydrocarbon microbubble could be found in the syringe, confirming that a saturated solution had been achieved. However, butane is a gas a room temperature and pressure, and a saturated aqueous phase cannot be achieved because butane vapor pressure exceeds atmospheric pressure. Instead, using FR or FR + agonist containing 1 atm O_2_ partial pressure in gastight glass syringes, butane drug solutions were prepared by repeated syringe headspace exchanges to yield solutions with gas partial pressures that were 90% butane and 10% O_2._

**Table 1:** Source, purity and physical properties of study compounds

Compound	CAS#	MW (amu)	P_vap_ (mmHg)	Solubility (M)	Carbon (#)	Volume (Å^3^)	Source	Purity (%)
Alcohols								
1-octanol	111-87-5	130.23	1.14 × 10^− 1^	9.0 × 10^− 3^	8	262	Alfa Aesar	>99
1-nonanol	143-08-8	144.25	4.07 × 10^− 2^	2.7 × 10^− 3^	9	290	Alfa Aesar	>99
1-decanol	112-30-1	158.28	1.48 × 10^− 2^	6.5 × 10^− 4^	10	317	Aldrich	>99
1-undecanol	112-42-5	172.31	5.10 × 10^− 3^	1.7 × 10^− 4^	11	344	Acros	98
1-dodecanol	112-53-8	186.33	2.09 × 10^− 3^	4.1 × 10^− 5^	12	372	TCI	99
1-tridecanol	112-70-9	200.36	8.07 × 10^− 4^	1.2 × 10^− 5^	13	400	Aldrich	97
1-tetradecanol	112-72-1	214.39	1.47 × 10^− 4^	2.1 × 10^− 6^	14	427	Fluka	>99
1-pentadecanol	629-76-5	228.41	1.27 × 10^− 4^	4.7 × 10^− 7^	15	454	Aldrich	99
Aldehydes								
nonanal	124-19-6	142.24	5.32 × 10^− 1^	2.3 × 10^−3^	9	289	Aldrich	95
decanal	112-31-2	156.27	2.07 × 10^−1^	9.8 × 10^−4^	10	316	Aldrich	98
undecanal	112-44-7	170.29	8.32 × 10^− 2^	4.2 × 10^− 4^	11	344	Aldrich	97
dodecanal	112-54-9	184.32	3.44 × 10^−2^	1.8 × 10^−4^	12	372	TCI	98
tridecanal	10486-19-8	198.34	1.46 × 10^−2^	8.2 × 10^−5^	13	399	TCI	98
tetradecanal	124-25-4	212.37	6.39 × 10^−3^	3.7 × 10^− 5^	14	427	TCI	98
heptadecanal	629-90-3	254.45	6.22 × 10^−4^	3.5 × 10^−6^	17	509	TCI	>97
octadecanal	638-66-4	268.48	3.00 × 10^−4^	1.7 × 10^− 6^	18	536	TCI	>95
docosanal	57402-36-5	324.58	2.02 × 10^−5^	9.1 × 10^−8^	20	646	Alfa Aesar	98
Alkanes								
butane	106-97-8	58.12	1.92 × 10^3^	1.4 × 10^−3^	4	156	Matheson	99.99
pentane	109-66-0	72.15	5.27 × 10^2^	4.3 × 10^−4^	5	184	Aldrich	>99
hexane	110-54-3	86.18	1.51 × 10^2^	1.2 × 10^−4^	6	211	Acros	>99
heptane	142-82-5	100.20	4.52 × 10^1^	3.1 × 10^−5^	7	239	Acros	>99
octane	111-65-9	114.23	1.42 × 10^1^	6.9 × 10^−6^	8	267	Acros	>99
nonane	111-84-2	128.26	4.63 × 10^0^	1.4 × 10^− 6^	9	294	Acros	99
decane	124-18-5	142.28	1.58 × 10^0^	2.6 × 10^−7^	10	321	Acros	>99
undecane	1120-21-4	156.31	5.64 × 10^−1^	4.2 × 10^−8^	11	349	Acros	99
tetradecane	629-59-4	198.39	2.85 × 10^−2^	6.1 × 10^−9^	14	431	Aldrich	>99
eicosane	112-95-8	282.55	1.40 × 10^−4^	2.9 × 10^−9^	20	596	Aldrich	99
Alkenes								
1-pentene	109-67-1	70.13	6.37 × 10^2^	1.4 × 10^−3^	5	176	Aldrich	99
1-hexene	592-41-6	84.16	1.88 × 10^2^	4.2 × 10^−4^	6	203	Aldrich	>99
1-octene	111-66-0	112.21	1.79 × 10^1^	2.6 × 10^−5^	8	258	Aldrich	98
1-nonene	124-11-8	126.24	5.77 × 10^0^	7.4 × 10^−6^	9	286	Aldrich	96
1-decene	872-05-9	140.27	1.92 × 10^0^	1.5 × 10^− 6^	10	313	Aldrich	>97
1-undecene	821-95-4	154.29	6.61 × 10^−1^	2.6 × 10^−7^	11	341	Aldrich	97
1-dodecene	112-41-4	168.32	2.34 × 10^− 1^	4.1 × 10^−8^	12	368	Aldrich	>99
1-tridecene	2437-56-1	182.35	8.56 10^−2^	5.8 × 10^−9^	13	396	Aldrich	96
Alkynes								
1-hexyne	693-02-7	82.14	1.35 × 10^2^	2.9 × 10^−3^	6	184	Aldrich	97
1-heptyne	628-71-7	96.17	4.35 × 10^1^	6.6 × 10^−4^	7	212	Acros	99
1-octyne	629-05-0	110.2	1.44 × 10^1^	1.9 × 10^− 4^	8	239	Acros	99
1-nonyne	3452-09-3	124.22	4.87 × 10^0^	3.9 × 10^− 5^	9	267	Aldrich	99
1-decyne	764-93-2	138.25	1.69 × 10^0^	7.9 × 10^−6^	10	294	Aldrich	98
1-undecyne	2243-98-3	152.28	6.05 × 10^−1^	1.4 × 10^− 6^	11	322	TCI	98
1-dodecyne	765-03-7	166.30	2.22 × 10^− 1^	2.4 × 10^−7^	12	349	TCI	98
Amines								
1-octadecanamine	124-30-1	269.51	4.88 × 10^−5^	1.3 × 10^−3^	18	546	TCI	97
1-eicosanamine	10525-37-8	297.56	8.96 × 10^−6^	2.7 × 10^−4^	20	601	Rambus	95
1-hexacosanamine	14130-10-0	381.72	8.91 × 10^−8^	2.3 × 10^−5^	26	766	ACC	98
1-octacosanamine	14130-12-2	409.77	2.21 × 10^− 8^	6.3 × 10^−6^	28	821	ACC	98
1-triacontanamine	66214-00-4	437.83	5.85 × 10^−9^	1.8 × 10^−6^	30	876	ACC	98
Cycloalkanes								
cyclopentane	287-92-3	70.13	3.14 × 10^2^	3.3 × 10^−3^	5	147	Aldrich	>99
cyclohexane	110-82-7	84.16	9.37 × 10^1^	1.0 × 10^−3^	6	176	Aldrich	>99.7
cycloheptane	291-64-5	98.19	1.99 × 10^1^	2.9 × 10^− 4^	7	206	Aldrich	96
cyclooctane	292-64-8	112.21	4.56 × 10^0^	7.2 × 10^−5^	8	236	Aldrich	>99
cyclodecane	293-96-9	140.27	4.47 × 10^−1^	3.2 × 10^−6^	10	295	Aldrich	95
cycloundecane	294-41-7	154.29	1.85 × 10^− 1^	5.7 × 10^−7^	11	324	Aldrich	95
cyclododecane	294-62-2	168.32	4.13 × 10^−2^	9.2 × 10^−8^	12	353	TCI	>99
Ethers								
dibutyl ether	142-96-1	130.23	7.10 × 10^0^	1.6 × 10^− 2^	8	277	Aldrich	99.3
dipentyl ether	693-65-2	158.28	1.00 × 10^0^	3.0 × 10^−3^	10	331	Fluka	>98.5
dihexyl ether	112-58-3	186.33	1.48 × 10^−1^	5.8 × 10^−4^	12	386	Aldrich	97
diheptyl ether	629-64-1	214.39	2.23 × 10^−2^	1.2 × 10^− 4^	14	442	TCI	98
dioctyl ether	629-82-3	242.44	4.53 × 10^−3^	2.4 × 10^−5^	16	497	TCI	98
didecyl ether	2456-28-2	298.55	8.08 × 10^−5^	1.2 × 10^−6^	20	606	TCI	98
diundecyl ether	43146-97-0	326.60	1.24 × 10^−5^	2.3 × 10^−7^	22	661	TCI	98

*CAS#* Chemical Abstracts Service number, *MW* molecular weight, *P*_*vap*_ vapor pressure at 25 °C, molar solubility in pure water at pH = 7, and molecular volume are calculated estimates (rather than measured values) referenced by SciFinder Scholar

### Data analysis

Drug responses were calculated as a percent change from the control (baseline) current responses as follows: $\% change=\frac{I_D-{I}_B}{I_B}$, where I_D_ and I_B_ are the current responses measured during perfusions with drug and without drug (baseline), respectively. Average current responses for each drug and channel were described by mean ± SEM. A positive current change indicated drug-induced positive allosteric modulation of the channel, whereas a negative current change indicated negative allosteric modulation (inhibition) of channel function. A drug-receptor cut-off response was defined as an absolute value change in current <10% from baseline that was statistically indistinguishable from zero using a two-tailed Student t-test. The log_10_ of the calculated solubility (log_10_S) for compounds immediately below and above the cut-off for each hydrocarbon functional group were used to determine the receptor cut-off. For each hydrocarbon, there was a “grey area” of indeterminate solubility effect between sequentially increasing hydrocarbon chain lengths. Mean solubility cut-offs were calculated as the average log_10_S for the least soluble compound that modulated receptor function and the most soluble neighboring compound for which no effect was observed. From this result, a 95% confidence interval for log_10_S was calculated for receptor solubility cut-offs.

## Results

**Fig. 1:**
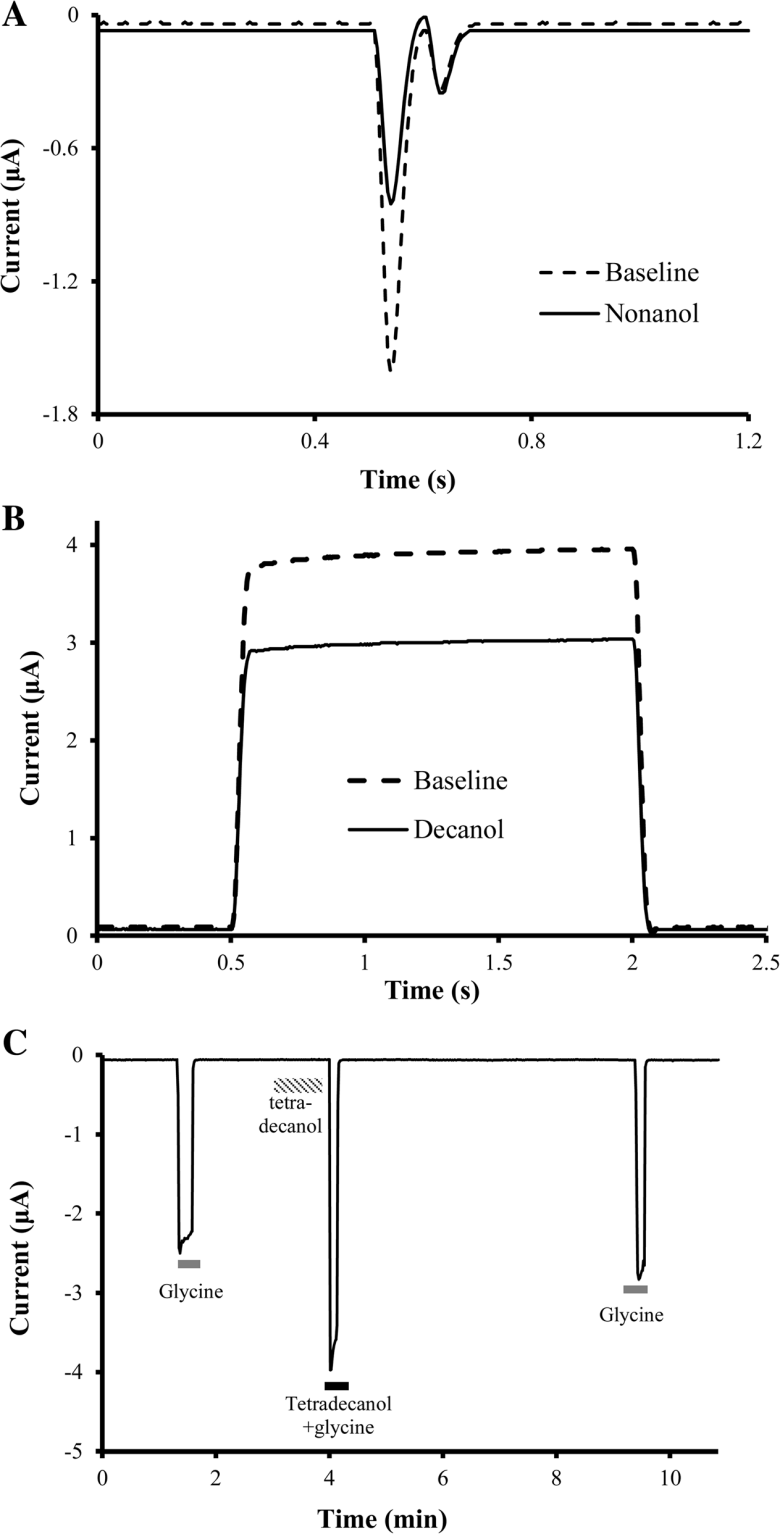
Sample tracings for (**a**) Na_v_1.2 channels, (**b**) TREK-1 channels, and (**c**) glycine receptors before and after alcohol exposure at saturated aqueous phase concentrations (Table 1). Whole cell current responses were qualitatively similar between both Na_v_ channels and between both K_2P_ channels. Electrophysiologic responses of GABA_A_ receptors during similar hydrocarbon exposure studies have been published elsewhere [6, 7]

Sample channel recordings are shown in Fig. 1, and hydrocarbon effects on each anesthetic-sensitive ion channel are summarized in Table 2. With increasing carbon additions to the ω-end or ring, all anesthetic-sensitive channels exhibited a cut-off effect, defined by <10% channel modulation, the smallest effect size that is reliably resolved given the given baseline variability allowed in these studies. All current modulation above this value was statistically different from baseline.

**Table 2:** Percent change ±SEM of whole-cell currents measured during two-electrode voltage clamp studies in response to administration of saturated concentration of each hydrocarbon (or 90% atm for butane)

Compound	Na_v_1.2	Na_v_1.4	TRESK	TREK-1	GABA_A_	Glycine
Alcohols						
1-octanol			74 ± 5 (5)*			
1-nonanol	−62 ± 7 (5)*	−48 ± 4 (6)*	41 ± 4 (5)*	−36 ± 2 (6)*		
1-decanol	−4 ± 1 (5)	− 35 ± 6 (6)*	2 ± 1 (6)	−28 ± 4 (6)*	322 ± 29 (5)*	
1-undecanol	−2 ± 1 (5)	− 4 ± 1 (5)		0 ± 2 (5)	81 ± 13 (5)*	
1-dodecanol					39 ± 6 (7)*	
1-tridecanol					2 ± 1 (6)	
1-tetradecanol				5 ± 3 (5)	4 ± 2 (5)	38 ± 6 (6)*
1-pentadecanol						−7 ± 2 (5)
Aldehydes						
nonanal	−51 ± 1 (6)*		−22 ± 6 (5)*		119 ± 29 (7)*	
decanal	−24 ± 3 (5)*	−31 ± 3 (6)*	−4 ± 2 (5)	−45 ± 3 (5)*		
undecanal	−6 ± 1(5)	−2 ± 1 (5)		−20 ± 2 (6)*		
dodecanal				− 5 ± 1 (5)		
tridecanal					20 ± 3 (7)*	
tetradecanal					5 ± 2 (9)	
heptadecanal						31 ± 2 (5)*
octadecanal						20 ± 3 (5)*
docosanal						1 ± 2 (7)
Alkanes						
butane	−20 ± 2 (7)*	−22 ± 2 (5)*	25 ± 5 (6)*	61 ± 9 (5)*	523 ± 68 (5)*	
pentane	−1 ± 1 (5)	− 2 ± 1 (5)	7 ± 1 (5)	−3 ± 2 (5)	221 ± 10 (7)*	
hexane		− 1 ± 0 (2)		4 ± 3 (4)	29 ± 5 (6)*	61 ± 5 (5)*
heptane		0 ± 1 (5)			−3 ± 1 (5)	
octane					− 1 ± 4 (2)	206 ± 21 (5)*
nonane						62 ± 18 (5)*
decane					5 ± 2 (2)	20 ± 3 (6)*
undecane						8 ± 2 (5)
tetradecane						5 ± 1 (5)
eicosane						9 ± 4 (2)
Alkenes						
1-pentene	−28 ± 2 (5)*	−45 ± 4 (5)*			351 ± 38 (6)*	
1-hexene	1 ± 1 (5)	0 ± 0 (6)	37 ± 7 (5)*	157 ± 13 (5)*	83 ± 8 (6)*	
1-octene			2 ± 1 (5)	1 ± 1 (5)	7 ± 3 (5)	
1-nonene					7 ± 4 (6)	
1-decene						
1-undecene						54 ± 7 (5)*
1-dodecene						−3 ± 3 (5)*
1-tridecene						1 ± 1 (5)
Alkynes						
1-hexyne	−44 ± 2 (5)*	−34 ± 2 (5)*	44 ± 5 (5)*		313 ± 23 (6)*	
1-heptyne	−2 ± 4 (5)	−4 ± 2 (6)	29 ± 5 (7)*	55 ± 5 (4)*	78 ± 5 (7)*	
1-octyne		2 ± 1 (5)	5 ± 2 (5)	−1 ± 2 (9)	− 3 ± 1 (6)	
1-nonyne					6 ± 1 (4)	
1-decyne					7 ± 1 (6)	55 ± 14 (5)*
1-undecyne						223 ± 20 (6)*
1-dodecyne						3 ± 2 (5)
Amines						
1-octadecanamine	−15 ± 1 (6)*	−11 ± 1 (6)*	−23 ± 2 (6)*	−16 ± 1 (5)*	46 ± 5 (8)*	
1-eicosanamine	0 ± 0 (6)	− 2 ± 2 (5)	−1 ± 1 (7)	−2 ± 3 (5)	67 ± 7 (6)*	
1-hexacosanamine			0 ± 2 (6)	−1 ± 3 (6)	1 ± 2 (6)	
1-octacosanamine						26 ± 3 (7)*
1-triacontanamine						2 ± 1 (6)
Cycloalkanes						
cyclopentane	−45 ± 4 (5)*	−11 ± 1 (6)*	63 ± 9 (5)*	68 ± 13 (5)*	93 ± 8 (7)*	
cyclohexane	−1 ± 1 (5)	−2 ± 2 (5)	1 ± 1 (6)	63 ± 6 (6)*	321 ± 17 (5)*	
cycloheptane	0 ± 1 (6)		0 ± 1 (5)	−4 ± 2 (6)	6 ± 3 (5)	
cyclooctane						
cyclodecane						22 ± 3 (5)*
cycloundecane						3 ± 2 (5)
cyclododecane						0 ± 1 (5)
Ethers						
dibutyl ether	−33 ± 3 (5)*	−44 ± 5 (5)*		50 ± 9 (6)*	234 ± 24 (5)*	
dipentyl ether	1 ± 1 (5)	1 ± 0 (5)	20 ± 2 (8)*	65 ± 6 (6)*	111 ± 9 (7)*	
dihexyl ether		0 ± 1 (5)	2 ± 1 (6)	3 ± 2 (6)	13 ± 1 (5)*	143 ± 10 (5)*
diheptyl ether		−1 ± 2 (2)			−1 ± 4 (5)	74 ± 8 (6)*
dioctyl ether					1 ± 3 (5)	95 ± 8 (5)*
didecyl ether						28 ± 4 (6)*
diundecyl ether						2 ± 2 (7)

Positive changes indicate drug potentiation of channel function, and negative changes indicate drug inhibition of channel function. Drug-induced changes ≥10% in magnitude in either direction that are significantly greater than zero (**not** a cut-off response) are indicated by an asterisk. The number of oocytes studied for each channel and drug combination is shown in parentheses

**Fig. 2:**
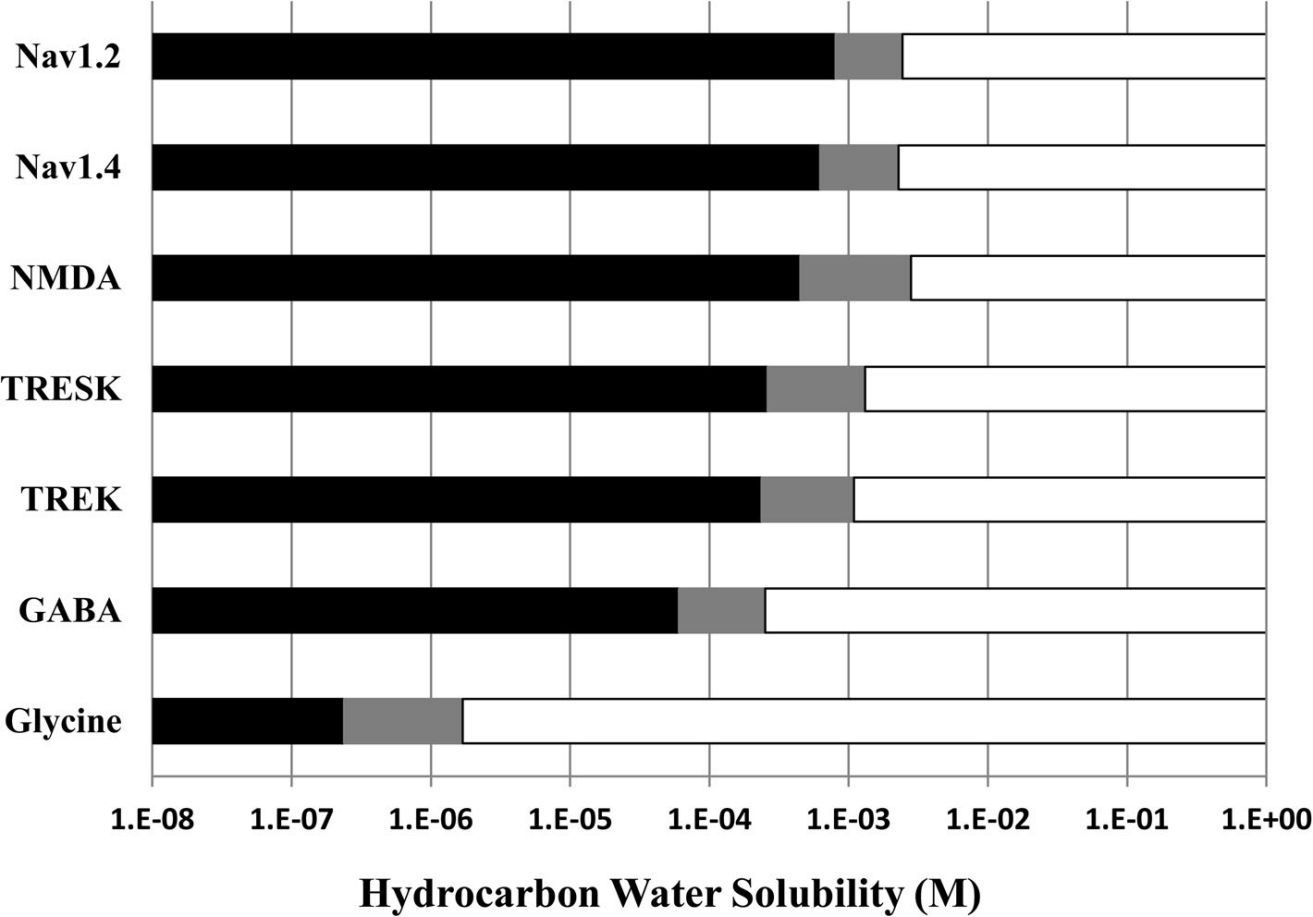
Summary of ion channel response as a function of hydrocarbon molar water solubility. Hydrocarbons that modulate ion channel function are indicated by a white bar. Hydrocarbons that did not affect whole cell currents for an ion channel are indicated by a black bar. The grey bar represents the 95% confidence interval around the mean hydrocarbon molar water solubility cut-off value for each ion channel

The mean hydrocarbon molar water solubility cut-off value and 95% confidence interval range for each ion channel is as follows (Fig. 2): Na_v_1.2 = 1.4 mM (0.81 mM–2.4 mM); Na_v_1.4 = 1.2 mM (0.62 mM–2.3 mM); TRESK = 0.59 mM (0.26 mM–1.3 mM); TREK-1 = 0.51 mM (0.24 mM–1.1 mM); GABA_A_ = 120 μM (60 μM–250 μM); Glycine = 0.63 μM (0.24 μM–1.7 μM). Loss of ion channel modulation generally occurred in this same order for all organic compounds, with some clustering of cut-offs for the Na_v_ and K_2P_ at similar molar water solubility values.

**Fig. 3:**
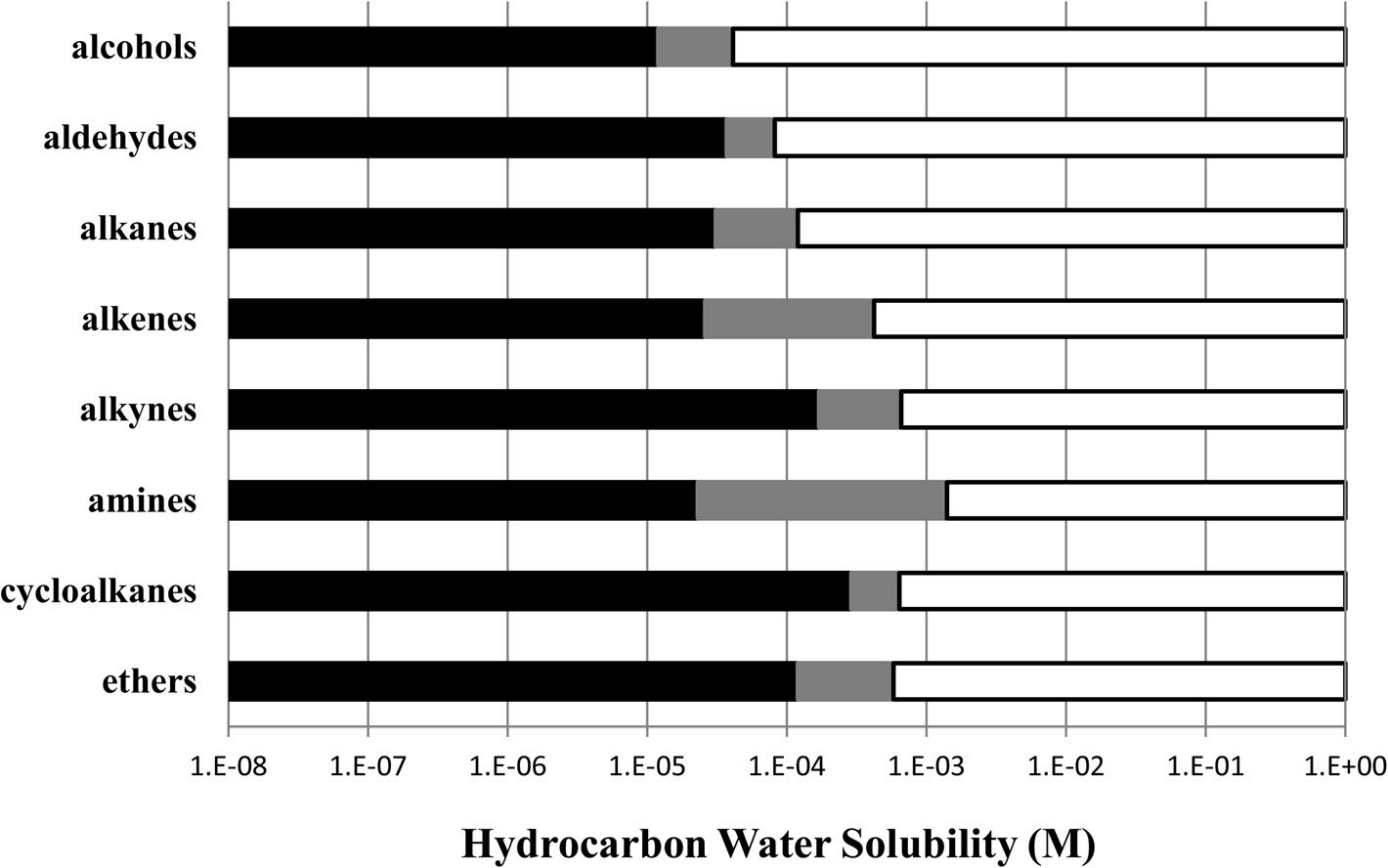
GABA_A_ receptor current potentiation (white bars) and absent whole cell current effects for eight different organic classes graphed as a function of the calculated hydrocarbon molar water solubility. The grey bars represent hydrocarbon solubility ranges within each organic class for which GABA_A_ receptor modulation was not evaluated. Cut-off values are clustered between 6.0 × 10^− 5^ and 2.5 × 10^− 4^ M

**Fig. 4:**
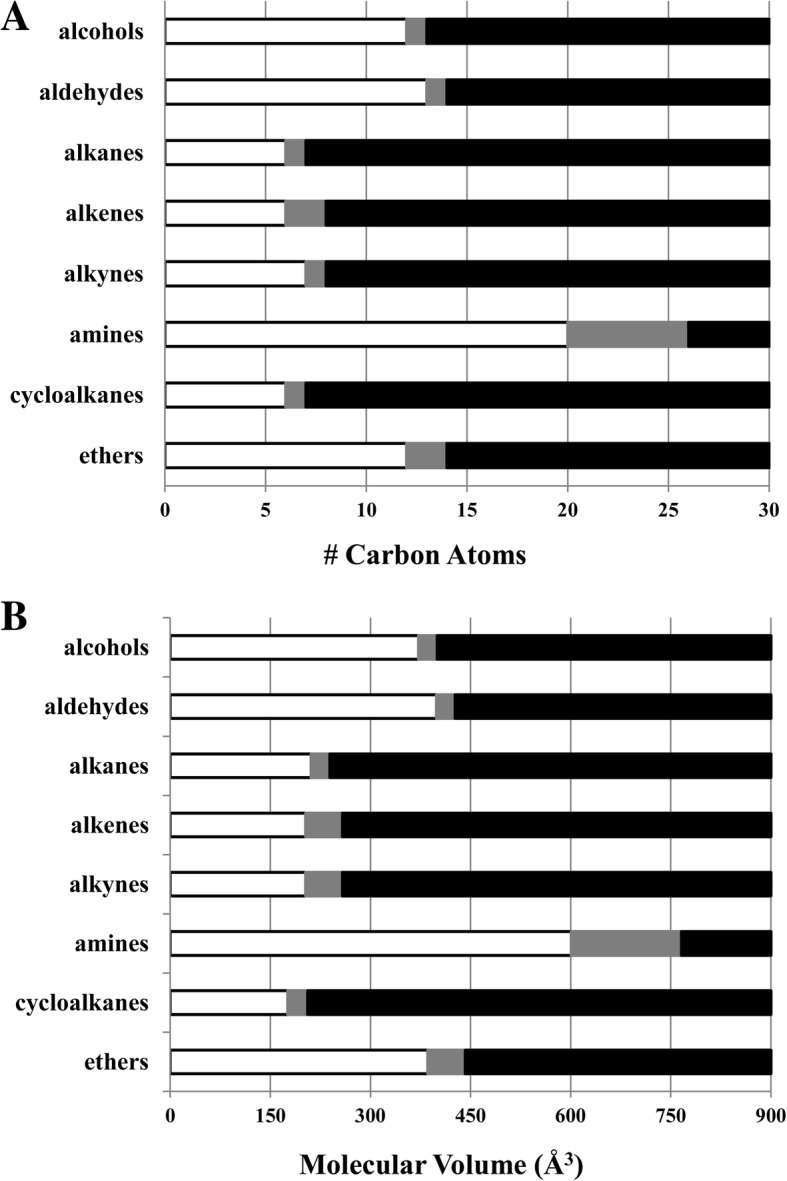
Graph of GABA_A_ receptor modulation as a function of the number of carbon atoms in a molecule (Panel **a**) and as a function of hydrocarbon molecular volume (Panel **b**). White and black bars indicate carbon numbers (Panel **a**) and molecular volumes (Panel **b**) associated with receptor potentiation and absent receptor modulation, respectively. Grey bars indicate regions for which hydrocarbon response data is not available. No pattern of consistent cut-off values associated with hydrocarbon chain length or molecular volume is evident

For each ion channel, the calculated molar water solubility was the only physical-chemical property examined that was associated with similar cut-off values between different hydrocarbon functional groups. An example of this is shown in Fig. 3 in which the grey indeterminate bars that separate known regions of positive and absent modulation are clustered around a relatively narrow molar water solubility cut-off range. Other channels, when similarly graphed as a function of hydrocarbon molar water solubility, show qualitatively similar results but with clustering around each channel’s own distinct molar water solubility cut-off value. In contrast, GABA_A_ receptor cut-off effects are not associated with the number of molecular carbon atoms or molecular volume of the hydrocarbon (Fig. 4), and these results mirror those of the other anesthetic-sensitive ion channels studied here.

Both Na_v_ channels were inhibited by all of the hydrocarbons tested above their respective cut-off values, whereas both of the ligand-gated ion channels (GABA_A_ and glycine receptors) were potentiated by all compounds above their respective cut-off values (Table 2). However, the K_2P_ channels were differently affected by compounds with different organic functional groups. Both were positively modulated by alkanes, alkenes, alkynes, cycloalkanes, and ethers, and both were negatively modulated by aldehydes and primary amines. However, whereas primary alcohols potentiated TRESK currents, they inhibited TREK-1 currents.

As increasing hydrocarbon chain length decreased molar water solubility to values close to a channel cut-off value, there was commonly a decrease in the magnitude of current potentiation or inhibition (Table 2). Notable exceptions to this pattern are cyclohexane with GABA_A_ receptors and undecyne with glycine receptors. Otherwise, there appeared no obvious relationship between hydrocarbon molar water solubility and either the magnitude or direction of receptor modulation.

## Discussion

As shown previously for NMDA receptors, all of the anesthetic-sensitive ligand-gated, voltage-gated and leak channels examined in this study exhibited cut-off effects for each class of organic compounds, and these cut-offs were associated with the calculated molar water solubility of the hydrocarbon. The cut-offs occurred in a predictable order, with Na_v_ channels K_2P_ channels, and GABA_A_ receptors cut-offs all clustered within roughly one order magnitude of saturated drug concentrations. In contrast, the glycine receptor cut-off was associated with drug molar water solubility values over two orders of magnitude lower. If previously determined NMDA receptor results are included [6], cut-off responses proceed in order of decreasing hydrocarbon solubility as follows: Na_v_1.2 ≈ Na_v_1.4 ≳ NMDA ≳ TRESK ≈ TREK-1 > GABA_A_ >> glycine (Fig. 2).

Hydrocarbon cut-off responses in the present study, defined by <10% effect, confirm data available in published literature. Horishita and Harris [13] found that the ability of primary alcohols to modulate Na_v_1.2 channels was lost between 1-octanol and 1-decanol, and is consistent with our finding of a cut-off between 1-nonanol and 1-decanol for this channel. Likewise, Peoples and Weight [14] observed GABA_A_ receptor potentiation with primary alcohol chains up to 1-dodecanol and a cut-off effect at 1-tridecanol and beyond, exactly where this cut-off was observed in the present study. The confidence interval for the GABA_A_ receptor molar water solubility cut-off from this study also encompasses the calculated solubility cut-off values for substituted benzene and phenolic compounds [7].

Discrete water solubility-associated cut-off effects may be a common feature of all inhaled anesthetic-sensitive ion channels. Conventional volatile and gas anesthetics immobilize individuals at high aqueous phase concentrations [15] suggesting that they engage in low-affinity binding with target receptors. We postulate that these conventional anesthetic-receptor interactions mirror experimental hydrocarbon-receptor interactions, and we propose a molar water solubility hypothesis to describe non-specific drug binding to low-affinity and amphipathic allosteric sites on proteins. Before a ligand can alter protein function, it must first bind at a site capable of inducing a change in protein function. In order to bind, water molecules must be removed from the hydration shell surrounding the drug, and water molecules within the amphipathic protein pocket must be displaced. The ease with which the water is displaced from the protein pocket is described by its dissociation constant (K_d_). Water that is only weakly bound to amino acid side chains within the protein pocket would have a high K_d_ and require fewer hydrocarbon molecules to successfully compete for access to this allosteric site. Consequently, this protein would have a lower molar water solubility cut-off. In contrast, water that is tightly bound within the pocket would have a low K_d_, and therefore a high hydrocarbon concentration would be necessary to compete for access to this site. Such a protein would exhibit a high drug molar water solubility cut-off. It is possible that ion channels may have more than one amphipathic allosteric site for low-affinity interactions with inhaled anesthetics or other hydrocarbons [16–18]; however the methods employed in the present study would have identified receptor cut-off effects based only on the amphipathic allosteric site with the highest water K_d_ in that receptor and which would therefore exhibit the lowest hydrocarbon molar water solubility cut-off effect among all modulatory sites. Other lower water K_d_ sites could be important for binding and modulation by more soluble hydrocarbons and inhaled anesthetics when present at sufficient aqueous concentrations.

A second explanation for hydrocarbon molar water solubility cut-offs is possible. Desolvation of water molecules around the ligand is necessary for protein binding to occur. However, if the difference in free energy between the solvated protein-drug complex and the separated solvated protein and solvated drug is too great, then drug binding will be energetically unfavorable and no modulation of protein function will occur [19]. Larger hydrophobic drug ligands are surrounded by larger and more rigid water shells for which desolvation may be associated with greater enthalpy. The strength of water complexes around the ligand might increase to the point that drug receptor binding—and thus drug-receptor modulation can only occur if there remains sufficient entropy to overcome increased enthalpy of binding [20].

In either case, a molar water solubility hypothesis is not predicated on any particular molecular size, shape, polarity, functional groups, or atomic arrangement of the drug for low affinity interactions to take place. Indeed, the diversity of conventional anesthetics and other hydrocarbons capable of modulating a single anesthetic-sensitive ion channel, as well as the relatively minor effect differences produced by dug enantiomers [21], suggest that ligand structure itself is not crucial for binding. However, ligand structure does determine the magnitude and type of modulation for the protein it binds. Whether there is inhibition versus potentiation of K_2P_ channel currents, for example, will depend on the functional group of the hydrocarbon ligand (Table 2). Modulation magnitude also differed between functional groups; inhibition of Na_v_1.4 channels was approximately four times greater for alcohols, alkenes, and ethers than for amines or cycloalkanes. The importance of structural elements within receptor binding pockets has also been demonstrated through mutation studies that confer resistance to conventional anesthetic or other hydrocarbon modulation [22–24], although it is unknown whether some of these changes may also have altered the water K_d_ or drug desolvation enthalpy that, in turn, could have affected the ability of the drug to bind the allosteric pocket.

The hydrocarbon molar water solubility cut-off value for each channel is expressed as confidence intervals, and certain error is inherent in their measurement. Butane, the smallest n-alkane studied here, is a gas at room temperature and pressure, and therefore cannot be studied at a saturated aqueous phase concentration under normobaric conditions unlike the other liquid and solid hydrocarbons. All receptors were nonetheless modulated by 90% atm of butane, but had current modulation not been observed, a true cut-off could have been inferred at this submaximal concentration. Furthermore, carbon additions to the ω-end of the hydrocarbon chain produce discrete, non-continuous changes in molar water solubility. For each series of functional groups, there is a range of solubility values that lie between the C_N_ modulating hydrocarbon and the C_N + 1_ cut-off hydrocarbon where the receptor effect is unknown. Most important, however, is the reliance on calculated solubility values for hydrocarbons in pure water at 25 °C and pH = 7.0 rather than measured solubility values under study conditions with a 250 mOsm electrolyte solution at 22 °C and pH = 7.4. Both accurate measurement and accurate prediction of solubility values are challenging for extremely hydrophobic compounds or for large or complex molecules with multiple functional groups. To limit this problem, only simple aliphatic compounds with the functional group on the first carbon, or central and symmetrical in the case of the dialkyl ethers, were studied. Even so, increasing hydrocarbon chain length is frequently accompanied by greater divergence between calculated and measured water solubility values [25, 26].

Whole cell current cut-off responses were measured using hydrocarbons at saturated aqueous concentrations. This was done to ensure that each cut-off was independent of any particular endpoint (e.g., amnesia, unconsciousness, or immobility). Lack of receptor modulation at a saturated hydrocarbon concentration implies absent modulation at a lower pharmacologic concentration, including concentrations relevant to anesthetic endpoints. Anesthetic efficacy is in mammals is unknown for many, but not all, of the hydrocarbons tested. Primary alcohol anesthetic potency increases with increasing carbon chain length from methanol to dodecanol, after which further carbon additions do not produce anesthesia at all [27]. This anesthetic cut-off corresponds to the alcohol molar water solubility cut-off for GABA_A_ receptors (Table 2). However, a general anesthesia cut-off effect has been reported to occur with n-alkanes and dialkyl ethers having around 11-to-15 or more carbon atoms [28] and cycloalkanes having eight or more carbons atoms [29]. These molecules are far longer and have molar water solubility values far lower than occur with the GABA_A_ receptor cut-off. Although observed anesthetic effects might be due to glycine receptor modulation, high affinity effects on one or more other anesthetic-sensitive receptors, or even systemic toxicity, it seems very possible that anesthetic effects could be the result of potent alcohol metabolites produced by oxidation of alkanes, cycloalkanes, and ethers by cytochrome P450 enzymes [30, 31]. Since the hydroxyl group confers greater molar water solubility, primary alcohols have receptor cut-offs at longer chain lengths than either alkanes or cycloalkanes or dialkyl ethers. These long-chain alcohol metabolites are also much more potent general anesthetics than their parent compounds [32], so even tiny quantities can have narcotic effects. Identifying simple parallels between in vitro receptor cut-offs and in vivo anesthetic cut-offs thus may be complicated for certain classes of organic compounds.

Channel studies were conducted using a reductionist biological system. However, in vitro electrophysiologic responses conducted at room temperature for relevant anesthetic-sensitive ion channels in oocytes seem to correlate with anesthetic potency in animals [33–35]. Likewise, the hydrocarbons studied in Table 1 administered in vivo would be expected to similarly modulate ion channels; in the case of drugs below the solubility cut-off, no in vivo modulation would be expected at all.

Finally, the molar water solubility hypothesis could offer practical applications to the development of new and novel inhaled anesthetic agents. Conventional volatile anesthetics bind promiscuously to a variety of cell proteins, but not all of these receptor interactions are essential to their ability to produce general anesthesia. For example, NMDA receptors contribute to immobilizing actions of inhaled anesthetics able to inhibit their function, but experimental inhaled anesthetics can still be immobilizers without producing NMDA receptor inhibition [36]. Since there is nearly a 10-fold separation in the hydrocarbon molar water solubility cut-off effects between NMDA versus GABA_A_ receptors, a volatile anesthetic might be modified to target calculated aqueous solubility values within this range to confer selectivity against higher cut-off NMDA receptors while preserving activity at lower cut-off GABA_A_ receptors. With sufficient GABA_A_ receptor potentiation and contributions of lower cut-off receptors, such an agent might retain immobilizing potency but lose adverse effects associated with the modulation of higher cut-off receptors. Consequently, molar water solubility could be key to identifying volatile anesthetics with new molecular mechanisms of action and improved pharmacodynamic profiles.

## Conclusions

Anesthetic-sensitive ion channels and receptors all appear to exhibit allosteric cut-off effects associated with drug molar water solubility. These results support the Molar Water Solubility Hypothesis mechanism for non-specific protein interactions with drugs of varied sizes and shapes administered at relatively high aqueous phase concentrations, such as occurs with inhaled anesthetic agents. Low-affinity binding at allosteric sites may occur following successful displacement of water by a hydrocarbon molecule (or portion thereof). However, when a hydrocarbon has a molar water solubility below the allosteric cut-off for a channel, as likely determined by the dissociation constant for water at the allosteric site, that hydrocarbon cannot be delivered at a concentration sufficient to outcompete the water for binding site access. In addition to describing how anesthetics of various sizes and shapes may be able to modulate functions of different anesthetic-sensitive ion channels and receptors, the Molar Water Solubility Hypothesis and order of receptor cut-offs offers a unique tool for the development of new inhaled anesthetic agents. By changing substituents on molecules to decrease their molar water solubility, it may be possible to generate novel inhaled anesthetics that are selective against some of the ion channels or receptors normally modulated by conventional volatile anesthetics.

## Abbreviations

GABA_A_: γ-aminobutyric acid receptor type A
Na_v_1.2: Voltage-gated sodium channels type II
Na_v_1.4: Voltage-gated sodium channels type IV
NMDA: N-methyl-d-aspartate
TREK-1: TWIK-related potassium channel type I
TRESK: TWIK-related spinal cord potassium channel
